# Non-communicable disease governance in the era of the sustainable development goals: a qualitative analysis of food industry framing in WHO consultations

**DOI:** 10.1186/s12992-020-00611-1

**Published:** 2020-08-26

**Authors:** Kathrin Lauber, Rob Ralston, Mélissa Mialon, Angela Carriedo, Anna B. Gilmore

**Affiliations:** 1 Tobacco Control Research Group, University of Bath, Bath, UK; 2 SPECTRUM Consortium, Edinburgh, UK; 3 Global Health Policy Unit, School of Social and Political Science, University of Edinburgh, Edinburgh, UK; 4 School of Public Health, University of São Paulo, São Paulo, Brazil; 5 World Public Health Nutrition Association, London, UK

**Keywords:** Commercial determinants of health, Non-communicable diseases, Corporate political activity, Global health governance, Food industry

## Abstract

**Background:**

The UN system’s shift towards multistakeholder governance, now embedded in the Sustainable Development Goals (SDGs), invites a broad range of actors, including the private sector, to the policymaking table. Although the tobacco industry is formally excluded from engagement, this approach provides opportunities for other unhealthy commodity industries to influence the World Health Organization’s (WHO’s) non-communicable disease (NCD) agenda. Focusing on the food industry, this research maps which actors engaged with WHO consultations, and critically examines actors’ policy and governance preferences as well as the framing they employ to promote these preferences in the global context.

**Methods:**

All written responses from food industry actors to publicly available NCD-relevant WHO consultations held between September 2015 and September 2018 were identified, totalling forty-five responses across five consultations. A qualitative frame analysis was conducted to identify policy positions expressed by respondents, as well as arguments and frames used to do so.

**Results:**

Though no individual companies responded to the consultations, the majority of participating business associations had some of the largest multinational food corporations as members. Respondents overarchingly promoted non-statutory approaches and opposed statutory regulation and conflict of interest safeguards. To this purpose, they framed the food industry as a legitimate and necessary partner in policymaking, differentiating themselves from the tobacco industry and referencing a history of successful collaboration, while also invoking multistakeholder norms and good governance principles to portray collaboration as required. Respondents contrasted this with the limits of WHO’s mandate, portraying it as out of step with the SDGs and framing NCD decision-making as a matter of national sovereignty.

**Conclusion:**

We observed that the UN’s call for partnerships to support the SDGs is invoked to defend corporate access to NCD policy. This highlights the need for more cautious approaches which are mindful of the commercial determinants of health. Systematic opposition to regulation and to governance approaches which may compromise commercial actors’ insider role in global health by food industry actors shown here, and the strategic use of the Sustainable Development agenda to this purpose, raises questions about the value of collaboration from the perspective of international health agencies such as WHO.

## Introduction

Non-communicable diseases (NCDs) have become the leading cause of global morbidity and mortality, causing over 73% of deaths in 2017 [1]. Unhealthy diets are a major driver of NCDs [2] and the proliferation of ultra-processed food and beverage products (‘ultra-processed foods’ hereafter) [3] plays a key role in this epidemic [4–7]. Ultra-processed foods are highly profitable, and predominantly produced and marketed by transnational corporations [8]. Attempts to introduce policies aimed at decreasing the consumption of ultra-processed foods have encountered strong pushback from these corporations (summarised under ‘food industry’ for simplicity) [9–15]. The WHO has published a range of recommendations and strategies which address dietary NCD policy. With an initial focus on individual-level, or downstream recommendations such as the majority of the 2004 Global Strategy on Diet, Physical Activity and Health [16], these have gradually incorporated population-level, or upstream approaches, for example in the Global Action Plan for the Prevention and Control of NCDs 2013–2020 [17]. Nonetheless, prominent political documents such as Time to Deliver [18] have stopped short of recommending fiscal measures [19]. Within global health governance, such soft regulation fulfils an important normative role [20], particularly in the absence of legally binding mechanisms. Therefore, despite being non-binding in the dietary NCD space, WHO guidance has prompted a major industry response, as illustrated by the Sugar Association’s aggressive counter-campaign to the 2004 proposal to limit free sugar intake to under 10 % of daily caloric intake [21].

Overall, growing evidence that the political activity of unhealthy commodity industry actors poses a major barrier to addressing NCDs has led to calls for clear, transparent, and accountable governance mechanisms to safeguard policymaking from corporate influence [2, 22, 23]. Yet, global health governance, traditionally constrained to “institutions and processes of governance which are related to an explicit health mandate,” [24] has seen an increasing move towards more networked [25] governance which involves non-governmental actors such as civil society and the private sector [26]. With the exception of the tobacco industry, which has been formally excluded from participation in public health policymaking through Article 5.3 of the Framework Convention on Tobacco Control (FCTC) [27], the multistakeholder governance model actively invites private sector actors to participate [28–30]. While the beginning of this partnership-oriented era pre-dates the year 2000 [31], it was formalised with the introduction in 2015 of the UN SDGs [32], specifically SDG 17 which calls on member states to “[e]ncourage and promote effective pubic, public-private and civil society partnerships” [32]. However, there are concerns that there may be an inherent conflict between SDG 17 and SDG 3, which includes a target to reduce premature mortality from NCDs by one-third [32], by lending legitimacy to partnerships with the private sector without acknowledging the well-established notion that these can undermine public health goals when they involve industries dependent upon the sale of unhealthy commodities [33, 34].

Public health policy and governance are not determined purely by the *translation* of scientific evidence through value- and interest-free analysis: central questions of authority and power highlight their inherently political nature [35]. Drawing on the notion that such power may be employed in instrumental or discursive ways, meaning it can be exerted through actions or arguments [36], this study focuses primarily on the latter. We aim to gain insights into how food industry actors attempt to shape debates around NCDs at the global level, using submissions to WHO consultations on NCD policy and governance held after the adoption of the SDGs. In so doing, it addresses a key gap in the literature on food industry influence which hitherto has focused on sub-national [37, 38] and national [9–14] levels of governance, with the global level neglected. Specifically, we address three key questions:

1. Which food industry actors participated in recent WHO consultations on NCD policy and governance?
2. What are their positions on policy and governance tools?
3. How are these positions justified?

To explore the latter we use frame analysis, adopting a constructivist view of framing which is rooted in the notion that there are multiple ways we can make sense of the social world [39]. Policy actors may understand an issue in differing ways, with frames as “structures of belief, perception and appreciation” underlying distinct policy positions [40]. Policy frames, described as “weapons of advocacy” [41], have the potential to shape policy processes and outcomes [42]. Framing an issue and its solutions in a certain way provides an “ordering logic” [43] and allows actors to set the terms of the debate and legitimise certain solutions while precluding others [42–44]. This has implications not only for how these matters are addressed, but may also affect governance structures by defining who is, and who is not, a legitimate stakeholder [44, 45].

## Methods

### Data sources

We undertook a two-step process to identify food industry submissions to relevant WHO consultations on NCD policy and governance. First, in January 2019, we systematically searched the WHO website for consultations which met the following criteria: (a) they were global in scope, (b) covered diet-related NCD policy and/or governance, (c) responses were published in full on the WHO website, and (d) they were held in the three-year window after the SDGs were ratified (September 2015–September 2018). Second, we identified submissions representing food industry interests by first selecting all private sector responses (separate from public sector, academia, and civil society; categorised as such by WHO in all but one consultation) and then, from these, selecting responses made directly by a food corporation or a business association (BA) overtly representing food industry interests (see Table 1 below for definitions). BA submissions were only included if the organisation explicitly stated that they represented the food industry, or parts of it, in their consultation submission. We did consider the inclusion of BAs with food industry members who did not explicitly mention this but decided against it as their responses did not address dietary NCDs sufficiently. No submissions were excluded based on language as all were either in English or Spanish.

**Table 1:** Definitions

**Food industry:** Food corporations and their representative bodies. This includes corporations manufacturing foods and soft drinks (focus on ultra-processed foods but not exclusive), or forming a key part of their supply chain, and holding a financial interest in the sale of foods and soft drinks, including ingredient supply and processing, retail, and fast food restaurant organisations.	
**Business association (BA):** A not-for profit entity which represents the interests of its members which are commercial enterprises and/or national or other business associations.	
**Cross-industry BA:** A business association representing a range of industries, often with a national or regional focus (e.g. US Council for International Business).	
**Food industry BA**: A business association which, according to its own information, predominantly represents the food industry (see above for definitions). Sub-classified as food, beverage, retail, or supply chain where specified.	

### Analysis

#### Submitting organisations

BAs were classified as either food industry BAs or cross-industry BAs (see Table 1 for definitions). To gain an insight into the companies represented by the included BAs, and given our time constraints, we searched BAs’ websites to identify which of the four largest ultra-processed food corporations were members (using global packaged food and soft drinks market shares as a proxy [46, 47]): Coca-Cola, Mondelez, PepsiCo, and Nestlé. We used the most recent membership information as of March 2020. Where previous membership lists were available, changes during the study timeframe (starting September 2015) were noted. A list of all organisations in our sample with membership details can be found in Additional file 1.

#### Policy positions and frames

##### Policy positions

After initial familiarisation with the data, the lead author (KL) identified the positions taken by each submitting organisation on a range of issues (the coding framework can be found in Additional file 2). They were coded in an Excel spreadsheet for whether a specific policy (e.g. taxation, advertising restrictions) or governance tool (e.g. conflict of interest (COI) safeguards, business impact assessments) was discussed and, if so, whether the organisation supported or opposed it.

##### Frames

We conducted a frame analysis [39, 43] to explore food industry attempts to shape the discourse around NCDs in support of their policy positions and preferred governance approaches. Upon reading of a set of sample documents from each consultation, members of the research team (KL, RR, MM) agreed on an initial list of relevant frames as overarching categories. This list was refined and added to throughout the iterative analytical process. Arguments were coded inductively as the smallest unit of analysis and grouped under overarching frames. KL coded all submissions in English (*n* = 36) and AC coded those in Spanish (*n* = 7). Moreover, 20 % of the consultation submissions were double-coded by a second member of the research team (AC) and discussions with the wider team followed to reach agreement on the findings (agreement was not quantified) and to refine them. This analysis was conducted using NVivo 12 [48].

## Results

### Sample

Five relevant consultations were identified (see Table 2), attracting a total of 437 submissions, 20.8% (91) thereof from private sector actors and 10.3% (45) explicitly representing the food industry (Table 2). These forty-five submissions comprise our sample. The remaining private sector responses included submissions from other unhealthy commodity industries (e.g. alcohol), a range of other commercial actors, and think-tanks. Of the included consultations, four focused on providing NCD policy recommendations, including but not limited to dietary interventions, and the fifth focused on developing a tool to help Member States manage COI. Of the five consultations, the WHO Independent High-level Commission on NCDs was the largest in terms of responses and the Zero Draft Shanghai Declaration on Health Promotion the smallest. One submission by Alimentos y Bebidas de Chile (AB Chile) [49] was excluded from the qualitative analysis as it only contained the draft consultation document without comments [49]. Another submission by the US Council for International Business (USCIB) [50] merely expressed support for another organisation’s statement and was therefore interpreted as identical.

**Table 2:** Included WHO consultations and a breakdown of total responses showing private sector submissions and the overt food/beverage submissions included within these

Consultation title	Dates	Details	Total number of responses	Private sector responses	Responses representing food industry
*Web-based consultation of the WHO Independent High-level Commission on NCDs*	10–16 May 2018	Held following a technical consultation in March 2018 [51] to inform the WHO Independent High-level Commission’s report to the UN High-Level Meeting on NCDs in September 2018. Outcome: *Time to deliver* [18]	215	47	19
*Consultation on the Member State-led draft outcome document for WHO Global Conference on NCDs (Montevideo Roadmap)*	11–25 August 2017	Consultation linked to the WHO Global Conference on enhancing policy coherence to prevent and control NCDs in October 2017. Outcome: *Montevideo Roadmap 2018–2030 on NCDs as a sustainable development priority* [52]	97	17	8
*Safeguarding against possible conflicts of interest in nutrition programmes*	11–29 September 2017	Consultation on an approach for the prevention and management of COI in the policy development and implementation of nutrition programmes at country level, following a technical consultation [53]. Outcome: *Safeguarding against possible conflicts of interest in nutrition programmes* [54].	44	14	12
*Updating Appendix 3 of the WHO Global NCD Action Plan 2013–2020*	25 July–1 September 2016	As requested at the 69th WHA, Appendix 3 of the WHO Global NCD Action Plan 2013–2020, a menu of policy options and cost-effective interventions for NCD prevention and control, was updated. This formed the basis of the WHO *Best Buys* [55].	64	9	4
*Zero draft Shanghai Declaration on Health Promotion*	20 May–15 October 2016	Consultation on a draft considered at the 9th Global Conference on Health Promotion in November 2016 which aimed to position health promotion as fundamental to Sustainable Development. Outcome: *Shanghai Declaration on promoting health in the 2030 Agenda for Sustainable Development* [56].	17	4	2
*Total numbers*			**437**	**91** ***(20.8%)***	**45** ***(10.3%)***

#### Submitting food industry organisations

**Fig. 1:**
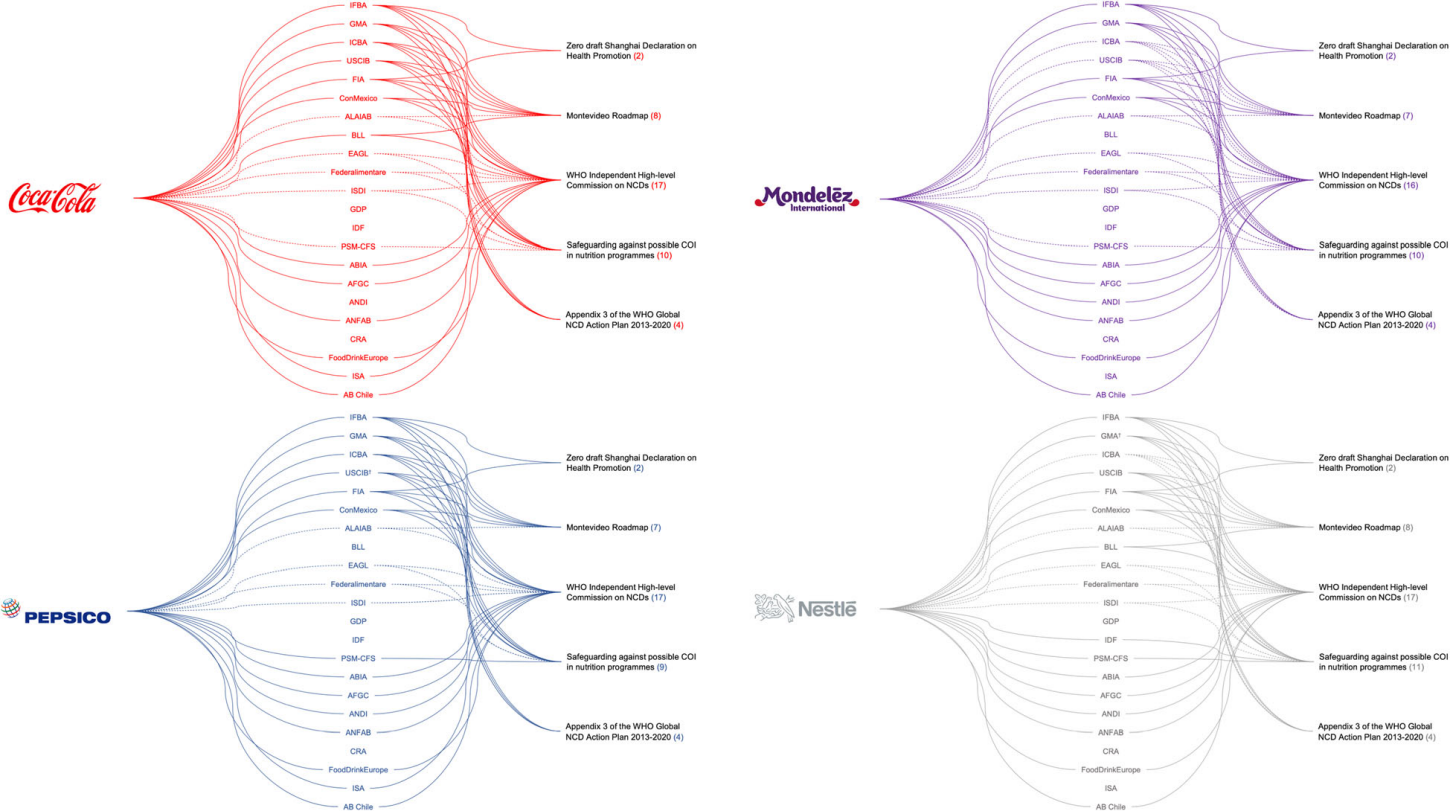
Food industry business associations and their membership links to the four largest, multinational ultra-processed food corporations. This figure shows food industry BAs who participated in the included consultations and their links to the four largest packaged food and soft drinks corporations (based on Euromonitor data for packaged food [46] and soft drinks [47]). Dashed lines between companies and BAs indicate an indirect link through a member organisation (* = ‘umbrella’ BA without direct corporate members). † indicates that a company was a member for at least part of the study period (Sept 2015 – Sept 2018) but has since left. More information in Additional file 1. This visualisation was created using PowerPoint [58]

No individual food companies submitted responses to any of the consultations; all twenty-two food industry actors represented across the forty-five submissions were BAs. Nineteen specifically represented food and/or beverage companies, and three were cross-industry BAs representing a range of sectors including food companies. Figure 1 below shows that, through these BAs, some of the largest multinational food corporations were represented multiple times in every consultation in our sample. The vast majority of BAs had at least one of the four selected food corporations as members. As a number of BAs within our sample only represented other BAs rather than individual corporations, we also traced such indirect links (dashed lines in Fig. 1). The International Food and Beverage Alliance (IFBA) was the only BA to respond to all consultations, followed by a group of similarly active BAs which responded to four out of the five consultations identified: the International Council of Beverages Associations (ICBA), the Grocery Manufacturers Association (GMA, now Consumer Brands Association [57]), USCIB, and Food Industry Asia (FIA).

### Positions on NCD policy and preferred governance approaches

Though expressing supporting for public health aims, food industry respondents did not support the idea of introducing new policies where they took a position on regulatory interventions such as taxation, marketing restrictions, or mandatory labelling, with sugar-sweetened beverage (SSB) taxation receiving the staunchest opposition. Instead, respondents expressed support for existing policies or alternative approaches such as education and self- or co-regulation of advertising and labelling, in particular positioning voluntary reformulation as key to tackling NCDs. In the context of proposed regulation, several respondents advocated for strong consideration of cost to businesses when assessing policies, with two explicitly calling for business impact assessments [59, 60]. The latter have raised concern in public health circles for identifying a clear role for industry and its evidence in policymaking [61]. Every single organisation supported a multistakeholder governance approach, with four respondents additionally calling for the private sector to be included in WHO’s reporting on NCD progress. Moreover, COI safeguards proposed in the ‘safeguarding against possible COI in nutrition’ consultation were opposed consistently by the food industry (summary positions for each policy can be found in Additional file 3).

Not only did food industry respondents express similar policy positions but several submissions contained sections which were identical apart from minor edits. Substantial overlap between submissions of the following BAs was noted: 1.) GMA [62] and USCIB [63], 2.) Federalimentare [64] and the UN Committee on World Food Security’s Private Sector Mechanism (PSM-CFS) [65], 3.) FIA [66] and IFBA [67], 4.) Global Dairy Platform (GDP) [68] and International Dairy Federation (IDF) [69], and 5.) FoodDrinkEurope [70] and ICBA [71].

### Framing

In exploring how the policy positions and preferred governance approaches above were justified, we identified seven key frames (Table 3). The interlinking frames and arguments are mutually supportive and promote the positions on interventions and preferred governance approaches discussed above. Maintaining the food industry’s reputation as a necessary and legitimate actor in public health emerged as an overarching ambition distinguishable across actors and consultations.

**Table 3:** Policy and governance frames and arguments. The ‘NCD interventions’ category refers to arguments about what should be done to tackle NCDs. For simplicity, NCD interventions were grouped into two categories: (a) regulation and (b) self- and co-regulation (SR/CR). The former comprises regulatory interventions such as taxation, marketing restrictions and bans, and mandatory labelling. The latter comprises non-statutory or voluntary initiatives and public-private partnerships to address NCDs. ‘Broader governance’ encompasses issues pertaining to how decisions are made, who governs, and who is governed. This includes, for example, broader questions about the role of industry in policymaking

Key arguments in the context of:			
FRAME	***NCD interventions***	***Broader governance***	
*Complexity*	Regulation is too simplistic for the complex issue of NCDs	The complexity of NCDs necessitates collaboration with industry	
*Regulatory redundancy*	Enough regulation is already in place	Conflict of interest is sufficiently managed (through FENSA)	
	Industry is already regulating itself		
	NCDs can be sufficiently addressed through non-statutory interventions		
*Partnership*	Successful past collaboration justifies a partnership approach to NCD policy	Industry is a necessary partner in addressing NCDs	
		Industry is a legitimate actor in NCD policy	Food industry is different to tobacco (or alcohol) industry
			Food industry is an important stakeholder
			Risk of engagement does not justify exclusion
*Unintended consequences*	Regulation may not have the intended health benefit (or may be counterproductive)	Restricting industry engagement in policymaking risks undermining the fight against NCDs	
	Regulation may have unanticipated negative consequences		
*Evidence*	There is insufficient (good) evidence in support of regulation	Industry supports evidence-based policymaking	
	The existing evidence does not support regulation	Evidence-based policymaking requires the inclusion of all available evidence (including from industry)	
*Good Governance*		Good governance requires working with industry	
		Achieving policy coherence requires private sector input and alignment of health with economic agendas	
*Limited WHO mandate*		WHO is overstepping its mandate	
		Restricting industry engagement is incoherent with the multistakeholder approach (and the SDGs)	
		National sovereignty	National governments hold the primary responsibility for addressing NCDs
			Policy may infringe on national sovereignty
			Policy may be incoherent with national legal obligations

#### Complexity

The complex nature of obesity and related NCDs was invoked to dismiss regulatory approaches as too simplistic. The German Food Federation (BLL), for example, expressed the view that “regulatory measures like marketing restrictions are not suitable to solve the complex issue of NCDs” [72]. Diet was framed as too complex for the regulation of specific products such as SSBs to be effective or appropriate, drawing attention to non-dietary factors such as a lack of physical activity. Promoting a “holistic” [73–77] approach to dietary public health policy instead, such arguments were employed to make the case that all products, including those considered unhealthy, play a role in people’s diets. This placed the blame for unhealthy diets as well as the responsibility for a “balanced” [73, 78, 79] lifestyle on the individual, justifying the food industry’s promotion of individual- rather than population-level interventions. In framing the issue as complex, respondents also fed into the narrative of partnership approaches as the way forward, USCIB, for example, claims that “[g]iven the complex nature of nutrition and health, making progress on NCDs will require partnerships to be formed between policymakers and non-state actors with lines of communication remaining open” [63].

#### Regulatory redundancy

In opposing regulatory approaches, respondents framed regulation as inappropriate or unnecessary by arguing that existing (regulatory or other) measures were sufficient, the current voluntary actions have achieved positive results; and where an intervention was necessary, non-statutory, self- or co-regulation would be preferable or should at least have “its place in the policy mix” [80]. FIA and IFBA, for example, highlight self-regulation as “cost-effective”, “measurable”, “flexible”, and able to “quickly respond to societal concerns” [81, 82]. The legitimacy of non-statutory approaches was emphasised by invoking WHO’s previous guidance, with FIA for example pointing out that “self-regulation has formally been recognised as a means of implementing the policy objective of reducing the impact on children of the marketing of foods high in fat, sugar and salt in the 2010 WHO recommendations” [60, 81]. Further marketing restrictions were also opposed by one German [72, 83] and one Brazilian [84] respondent on the basis that sufficient regulation was already in place at country level. In a similar vein, a frequent argument against COI safeguards maintained that sufficient protections existed, often directly invoking the WHO’s Framework of Engagement with Non-State Actors (FENSA, [85]) [64–66], even though the proposed safeguards focused on member state management of COI whereas FENSA is an internal WHO guide [53].

#### Partnership

Respondents employed a partnership framing to convey a need for industry participation to adequately tackle NCDs and maintain that food industry actors have a right to participate. Portraying themselves as a part of the solution, food industry actors invoked the urgency of collaboration in order to tackle the NCD challenge. Highlighting the imbalance between available and required public resources to prevent and treat NCDs, respondents argued that the food industry is needed to fill such gaps: “Given the importance of tackling NCDs and the limited technical expertise and resources available in many countries, public-private engagement will be extremely important to better address the challenges of NCDs” [86].

A partnership framing and the use of language around ‘multistakeholder’ and ‘multisectoral’ approaches were also used to position the food industry as a legitimate policy actor with the right to participate. The idea of a shared purpose between industry and the public sector was employed to paint a picture of the industry as a partner, rather than an opponent or a bystander, in NCD policy. To highlight the food industry as a vital stakeholder, respondents emphasised its economic importance [59, 63, 87] and positioned it as a bridge between government and consumers, arguing that “businesses in the food sector have the closest direct relationships with consumers through their day to day food and diet choices” [88]. Ostensibly successful previous collaboration with governments and international organisations, as well as ongoing voluntary commitments, were cited to demonstrate that the food industry deserves a strong role going forward. Invoking the food industry’s “long and successful history of engaging on nutrition and health issues at national, regional, and global levels”, GMA added that “key public health initiatives, such as the development of salt reduction strategies, could not have been achieved without constructive engagement between health authorities and our industry” [59]. Respondents also explicitly differentiated the food industry from other sectors whose products are perceived as more harmful, such as alcohol and tobacco. In particular, food industry actors heavily protested any comparison or association with the widely denormalised tobacco industry, considering it “inappropriate for the WHO to compare the food and beverage industry to industries excluded from engagement with the WHO” [64, 65]. In a similar vein, respondents opposed the use of the term ‘health-harming’ on the basis that it would inappropriately equate their products with alcohol and tobacco. One respondent stated that although “high-fat, sugar and salt products should be consumed in a balanced manner, it would not be appropriate to classify such products as “health-harming”, presumably in the same category as tobacco products or alcohol beverages” [84]. While acknowledging a perceived risk from private sector engagement in some cases, COI was widely downplayed as manageable through disclosure and the notion of excluding actors due to COI was dismissed as “unfair” [64, 65].

#### Unintended consequences

Respondents questioned whether regulatory policies would have the intended benefit for public health and argued that they may have unintended negative effects. Food industry actors claimed that regulatory policies would not work, either because they are too narrowly focused (see ‘Complexity’) or because, as seen particularly in the context of SSB taxation, they would not have the desired effect of decreasing consumption of the target product or nutrient [74]. Conversely, the same respondents also warned against devastating impacts of SSB taxation on businesses, employment, the economy, or low-income groups. ICBA, for example, justified its opposition to SSB taxation by maintaining that “history has also shown that these types of tax initiatives are regressive, and are unlikely to actually affect obesity rates and thus non-communicable diseases” [74]. Closely linked to the partnership framing discussed above, food industry actors argued that the proposed COI safeguards may undermine efforts to reduce NCDs by, for example, depriving “member states of the knowledge, expertise, and resources of the private sector and put at risk programs that are helping member states achieve their own public health objectives” [64]. A number of respondents, for example GMA and USCIB in two near-identical submissions, explicitly presented non-engagement with industry as a threat to NCD policy, arguing that “it is essential that WHO support government engagement with private sector, including by helping governments understand missed opportunities and assess risk of not engaging” [59, 63].

#### Evidence

A central element to commercial actors’ arguments was evidence, including the principle of evidence-based policymaking. Regulatory policies were contested with claims that the evidence base is either insufficient in quantity or quality, or contraindicative (closely linked to ‘Unintended consequences’). Respondents questioned the evidence base underlying regulation, BLL for example asserted that “[a]dvertising plays – if it all – a minor role in influencing food choices (…). This has been shown in practice and scientific studies” [72] and ICBA questioned the link between SSBs and obesity, stating that “the overall weight of the scientific evidence on sugar and/or sugar-sweetened beverages show that they do not have a unique effect on body weight beyond their contribution to total calorie intake” [74]. An apparent lack of evidence was often put forward to justify delaying policy action. IFBA, for example, claimed that “further work is needed to assess the impact as well as the implementation approaches of fiscal measures before these are recommended” [79] and GMA argued that “there are significant evidence gaps in understanding how label format impacts consumer behaviour” [73]. The distinction between healthy and unhealthy foods itself, and the use of associated terms such as processed and health-harming was contested with claims that there is a lack of evidence supporting them. Widely pledging their support for evidence-based policymaking in principle, food industry actors also portrayed industry engagement as an important part of it, arguing, for example, that “[p]rivate sector engagement is, and has been, a key contributor to evidence-based policymaking at the WHO” [86]. More specifically, multiple submissions argued that evidence-based policymaking demands the consideration and inclusion of all evidence, including the industry’s own.

#### Good governance

The concept of good governance was invoked throughout the consultation responses, using the indeterminate but ubiquitous term to advocate a very specific approach to decision-making – a significant role for the private sector and emphasis on minimising the economic impact of health policies. Despite rhetorical alignment with accountable and transparent policymaking, respondents framed COI safeguards as incoherent with, or even “antithetical” [64] to, good governance. A number of US-based BAs explicitly made reference to “good regulatory practice”, which, according to USCIB, includes “transparent stakeholder consultation, cost/benefit analysis, and assessment of alternatives to regulation” [89]. Such arguments were closely tied to the promotion of economic impact assessment.

Furthermore, policy coherence, within the WHO and with the wider UN system, was emphasised by respondents in support of their positions and preferred governance approaches. The SDGs as well as the WHO’s own FENSA and publications on NCD policy were invoked to oppose COI safeguards in nutrition policy. FIA, for example, expressed its concern that the proposed safeguards “perhaps contradict the spirit of FENSA, as well as the WHO’s Global Strategy for the prevention and control of non-communicable diseases 2013-2020, the 2011 UN Political Declaration on NCDS, and the UN’s Sustainable Development Goals, specifically SDG 17” [66]. Correspondingly, industry engagement was positioned as essential for increasing policy coherence. The concept, often invoked with the aim of improving the alignment of other policy agendas with health, was reframed by Engaging America’s Global Leadership (EAGL) [90] as going “in both directions”, meaning that health ministries should also align their approaches with other policy areas such as “economic growth and development” [90].

#### Limited WHO mandate

Submissions questioned the political legitimacy of the WHO regarding its role within the UN system, its internal coherence, and issues of national sovereignty. Closely related to arguments around policy coherence mentioned above but instead conveying a sense of the WHO’s NCD agenda being subordinate to the wider UN’s Sustainable Development Agenda, the WHO’s restricted room for action was highlighted. The WHO Department of Nutrition’s attempts at developing guidance for addressing and managing COI in nutrition policy were framed as out of step with other WHO policies, the SDGs and the overall multistakeholder approach prominent within the UN system. Citing SDG 17 directly in several cases, respondents interpreted the Sustainable Development agenda as an imperative for partnership, suggesting that the WHO must comply with this approach. Similarly, the WHO was portrayed as an agency with a narrow focus and capability. For example, its expertise and authority on issues such as fiscal policy was questioned by actors opposing SSB taxation: “Offering such policy advice in a field – economics and fiscal policy – far from WHO’s expertise is not in our opinion a prudent course of action” [71, 91].

Respondents also emphasised national primacy in decision-making, arguing that questions such as whether to engage with industry in policymaking should not be answered by the WHO, but left to Member States. Correspondingly, and despite their non-binding nature, proposed COI safeguards were portrayed as potentially infringing on national sovereignty, and contravening Member States’ legal obligations: “Member States must observe their own legal and normative obligations to free and open governance to deliver regulations that meet public policy objectives” [92]. The US-based ICBA [93] and GMA [94] explicitly referred to protections for commercial speech as the potentially conflicting policy area. Furthermore, the primary responsibility and authority for tackling NCDs was attributed to national governments, implying that the WHO has a limited role to play. Emphasising that NCD policy should be driven by national priorities, respondents advocated flexible and non-restrictive, namely weaker, recommendations.

## Discussion

This study offers new insights into the ways in which food industry actors engage with WHO consultations. Firstly, it shows that this formal type of engagement takes place though BAs at this level, the vast majority of which represent the largest ultra-processed food manufacturers. Identical sections in submissions from different BAs indicate a degree of coordination even though these industry actors are competitors in their respective markets. Secondly, our research confirms existing evidence that food industry actors support non-statutory measures and largely oppose further statutory regulation. In terms of preferred governance approaches, respondents strongly backed a multistakeholder approach and, while acknowledging that COI needs to be managed, opposed the safeguards proposed by the WHO’s Nutrition department [54]. Third, we explored how industry actors framed the issue at hand to support their preferences. In addition to using a range of arguments common in national public health policy contexts, they invoked a range of malleable concepts, echoing language commonly used by commercial actors globally to oppose regulation and emphasise an ostensible need for the public sector to engage with the private sector in policy development.

Previous research using framing as a lens to explore food industry rhetoric in public health has focused mostly on obesity as an issue [38, 95] or specific policies such as SSB taxation [37, 96] and public-private initiatives [97]. Our research adds to this by exploring and connecting a broad spectrum from specific NCD interventions to questions around broader public health governance. In the context of interventions, our findings echo arguments which have been documented in the context of country-level public health policy debates, both by food companies and other unhealthy commodity actors. We confirm existing research from the national context which demonstrates that industry framing portrays public health issues as individual or lifestyle choices, and consequently interventions addressing individual responsibility, such as education, as the solution [13, 95, 98]. This framing also rules out policy solutions which target the environment rather than the individual, for example regulatory measures such as taxation and advertising restrictions [95]. Principles such as market justice, individual responsibility, and personal freedom are common across industry framing in dietary public health debates [95, 99] as well as other issues such as tobacco [36, 100, 101] and alcohol [43, 102]. Despite rhetorically aligning themselves with evidence-based policymaking, respondents overarchingly opposed regulatory in favour of non-regulatory policy options which independent research suggests are less effective [103, 104]. Complexity framing of public health issues has also been documented across unhealthy commodity industries [105]. Similarly, calls for the enforcement of existing policies or voluntary measures as opposed to the introduction of new policies are commonly observed across unhealthy commodity industries [102, 106], with the term ‘regulatory redundancy’ first introduced by tobacco control researchers [107]. Due to tobacco companies’ role as involuntary outsiders to public health policymaking, being likened to this industry appears to prompt heavy opposition in other commercial actors, a finding previously noted in the context of the alcohol industry [102].

Narratives which question the WHO’s legitimacy link into a broader discussion about its place within the UN system. Food industry organisations challenged the agency’s legitimacy and mandate, firstly by framing proposed restrictions on industry engagement as incoherent with good governance principles, the SDGs, and the internationally dominant multistakeholder approach, and secondly by questioning the WHO’s mandate on NCD policy in the context of regulation. This closely mirrors tobacco industry rhetoric in opposition to the WHO’s FCTC [100], indicating that some arguments may be consistent across unhealthy commodity industries on the global level. Moreover, arguments around national sovereignty were employed to shift decision-making away from the WHO where industry actors did not agree with the policies proposed. Such attempts at venue-shifting to other levels of governance where a policy may be easier to contest have been documented in the case of the tobacco industry’s campaign against standardised packaging [108]. Our analysis of submissions to WHO consultations highlights how political strategies constructed jurisdictional boundaries as a tactic to curtail the WHO’s mandate in NCD governance. Future research might draw on the theoretical framework of multi-level governance [109] to explore how political activities and messaging are coordinated globally, or to explore the strategic role of business associations as intermediaries between different levels of governance.

Food industry actors promoted their policy and governance approaches preferences using a range of what Smith calls ‘chameleonic ideas’ [110] which, vague and flexible, may have different meanings depending on how they are framed. Terms which are ubiquitous in the global health policy space, such as good governance and policy coherence, were invoked selectively by industry actors to promote a very specific brand of policymaking. For example, the malleable term ‘good governance’ was employed in consultation responses to promote a neoliberal view of policymaking, with a strong seat at the table for industry and severely limited space for government regulation. Similarly, the concept of policy coherence, often invoked to promote the inclusion of health in all policies, was used to suggest that, conversely, health policies should be adapted to accommodate economic concerns. Policy coherence was used selectively to support preferred governance approaches and oppose COI safeguards based on discrepancies with UN guidelines which encourage private sector engagement, but respondents were silent on policies which do the opposite, such as Article 5.3 of the WHO FCTC which excludes the tobacco industry from public health policymaking. While invoking the idea of complexity to push back against ostensibly simplistic regulatory measures that target one ingredient or type of product, food industry actors simultaneously conveyed a simplistic view of obesity and related NCDs which is deeply rooted in the notion of individual responsibility. In so doing, they failed to acknowledge its genuinely complex causes [111] and the need for comprehensive measures to address it.

Though no companies participated directly, the largest ultra-processed food producers were represented by the vast majority of the BAs which responded to the WHO’s consultations on NCD policy and governance. While it is not known how much input member companies had into the content of the submissions we analysed, we demonstrate that Coca-Cola, PepsiCo, Nestlé, and Mondelez were all represented multiple times in every consultation we examined. BAs generally appear to play a key role in coordinating food industry and wider commercial engagement at the global level, which could be partially explained by the fact that the WHO Executive Board may grant international BAs, but not individual corporations, Official Relations status [112]. Some of the BAs in our sample publicise an explicit focus on providing access to international institutions: USCIB, for example, claims on its website that “[n]o other American organization can provide comparable channels for business to interface with key inter-governmental bodies such as the United Nations” [113]. Similarly, IFBA, which responded to every single consultation covered in this study, was set up by CEOs of the major international food and beverage corporations in response to the 2004 Global Strategy on Diet, Physical Activity and Health, specifically to engage with WHO and other multilateral institutions [114]. Indeed, the prevalence of BAs in the global policy space also raises a potential tension with the WHO’s policy of non-engagement with the tobacco industry: even though some tobacco-linked entities were excluded from WHO consultations (e.g. the Philip Morris-funded Foundation for a Smoke-Free World) [115], a lack of transparent reporting nonetheless enabled business associations representing tobacco companies to participate (e.g. USCIB [116] and BLL [117]).

Our research echoes concerns by academics and advocates that the multistakeholder imperative within the UN system is being employed to sustain corporate access to public health policymaking [33, 34]. Specifically, SDG 17, which calls for multistakeholder partnerships, can be interpreted as a blanket appeal to collaborate with the private sector. As such, an open door for commercial actors (bar the exception of tobacco and arms) and the idea that societal complexity requires public-private collaboration not only appears to facilitate opposition to safeguards against undue influence in public health policy, it also implies a stronger role for industry self-regulation. With experts increasingly calling for population-level, regulatory interventions to tackle NCDs in the face of a global lack of progress [19, 118], industry framing of the issue promotes alternatives to regulation which may weaken such attempts at governing the commercial determinants of health. In contributing to our understanding of industry framing around preferred policies and governance approaches, our findings support non-industry actors involved in global-level policy debates to strengthen strategies to prevent industry interference, and to debunk or counter the identified arguments from a public health perspective. One area to explore is strategic counter-framing, for example by responding to arguments based on the corporate right to participate in global health governance with counter-framing rooted in the human right to health [119].

Our research has a number of limitations which must be acknowledged. Publicly available consultation responses, though easily accessible, grant only limited insights into political activity. Thus, while our analysis covers rhetoric used to try and influence policy and participation in consultations, further research should complement this by exploring the action-based influencing strategies used to disseminate and legitimate this narrative. Examples of other ways commercial actors may exert influence include the participation of food industry actors in WHO meetings, such as IFBA’s presence at the UN High-Level Meetings on NCDs [120, 121]. Though we focused solely on organisations overtly representing the food industry, it remains an important task for future research to explore arguments and activities of groups which are linked to the industry, for example financially or through interlocking directorates [122]. Building on our study which captures the overt industry narrative, establishing similarities and differences between the way linked groups frame the issue is a crucial next step. Lastly, while this is not something we attempted to do in this study, we support recent calls for more research on the impact of corporate political behaviour [123].

## Conclusions

In summary, our findings reveal how multistakeholder governance approaches in the wider UN system may be exploited by corporations attempting to maintain and expand their legitimacy in the NCD policy space, while constraining that of the WHO. Amid the tensions between the UN system’s efforts to democratise global health governance and the pro-industry norms underlying multistakeholder governance, a door has opened for commercial actors. Actors whose vast resources give them greater access than civil society enjoys to a space where their interests directly conflict with the public good. The SDGs, by embracing multistakeholder collaboration as the way forward, set a status quo which may undermine attempts to regulate the commercial determinants of health [124, 125]. Policymakers and institutions aiming to tackle NCDs globally are faced with important decisions on who they consult and collaborate with. Amidst a changing dynamic between *the governed* and *the governing*, the food industry is not only a vocal advocate for a prominent role of corporations in global health, but also a critic of the WHO as a leading force in NCD policymaking. Such attempts to undermine the agency’s position during ongoing reforms should be noted with concern. Lastly, current governance approaches do not appropriately address similarities in behaviour and rhetoric between unhealthy commodities industries, with tobacco excluded and others welcomed to the table. Considering the lack of progress under the existing multistakeholder paradigm, it may be time for a rethink.

## Supplementary information

**Additional file 1.** Business associations and their links to a purposive sample of four multinational food corporations. This file provides additional details about the business associations in our sample, the links to food corporations displayed in Fig. 1, and the evidence used to establish these connections.

**Additional file 2.** Coding framework used to map positions on policy and preferred governance approaches. This file describes in more detail the framework used to code the policy positions of consultation respondents in our sample.

**Additional file 3.** Summary of policy positions across all business associations in our sample. This file provides a quantitative overview of how many BAs in our sample supported or opposed a number of policies.

## Data Availability

The consultation responses analysed in this study are publicly available on the WHO website.

## Abbreviations

AB Chile: Alimentos y Bebidas de Chile
ABIA: Associação Brasileira da Indústria de Alimentos
AFGC: Australian Food and Grocery Council
ALAIAB: Alianza Latinoamericana de Asociaciones de la Industria de Alimentos y Bebidas
ANDI: Asociación Nacional de Empresarios de Colombia
ANFAB: Asociación Nacional de Fabricantes de Alimentos y Bebidas
BA: Business Association
BLL: German Federation for Food Law and Food Science
COI: Conflict of interest
CRA: Corn Refiners Association
EAGL: Engaging America’s Global Leadership
FCTC: Framework Convention on Tobacco Control
FIA: Food Industry Asia
GDP: Global Dairy Platform
GMA: Grocery Manufacturers Association (now Consumer Brands Association)
ICBA: International Council of Beverages Associations
IDF: International Dairy Federation
IFBA: International Food and Beverage Alliance
ISA: International Sweeteners Association
ISDI: Alianza Latinoamericana de Asociaciones de la Industria de Alimentos y Bebidas
NCDs: Non-communicable diseases
PSM-CFS: Private Sector Mechanism to the Committee on World Food Security
UN: United Nations
SDGs: Sustainable Development Goals
USCIB: United States Council for International Business
WHO: World Health Organization
