# Supplementary material for: Non-communicable disease governance in the era of the sustainable development goals: a qualitative analysis of food industry framing in WHO consultations
Source: Global Health. 2020 Aug 26;16:76. doi: 10.1186/s12992-020-00611-1 (PMC7448499; doi:10.1186/s12992-020-00611-1)
Supplement: Supplementary file 1 — Additional file 1. Business associations and their links to a purposive sample of four multinational food corporations. This file provides additional details about the business associations in our sample, the links to food corporations displayed in Fig. 1, and the evidence used to establish these connections. [file 12992_2020_611_MOESM1_ESM.docx]

**Additional file 1: Business associations and their links to a purposive sample of four multinational food corporations**

**Table A1***:* This table indicates whether any of the four largest packaged food and soft drink companies are members of the business associations in our sample (includes subsidiaries). A ✓ means that the company is a direct member, a (✓) signifies an indirect link through another organisation (supporting evidence is linked). We used the most recent available information in March 2020. Past membership if it changed after consultation submissions were made.

| Name | Description | Membership | | | |
| --- | --- | --- | --- | --- | --- |
|  |  | *Coca-Cola* | *PepsiCo* | *Nestlé* | *Mondelez* |
| International Food and Beverage Alliance (IFBA) | Association of the twelve largest food and soft drink companies which focuses on the global level of policymaking and holds special consultative status with the UN’s Economic and Social Committee. | [✓](https://web.archive.org/web/20190604141811/https:/ifballiance.org/) | [✓](https://web.archive.org/web/20190604141811/https:/ifballiance.org/) | [✓](https://web.archive.org/web/20190604141811/https:/ifballiance.org/) | [✓](https://web.archive.org/web/20190604141811/https:/ifballiance.org/) |
| ConMexico | Mexican trade association bringing together food, beverage (alcoholic & non-alcoholic), household, and personal care sectors. | [✓](https://web.archive.org/web/20191219192123/https:/www.conmexico.com.mx/) | [✓](https://web.archive.org/web/20191219192123/https:/www.conmexico.com.mx/) | [✓](https://web.archive.org/web/20191219192123/https:/www.conmexico.com.mx/) | [✓](https://web.archive.org/web/20191219192123/https:/www.conmexico.com.mx/) |
| Food Industry Asia (FIA) |  | [✓](https://web.archive.org/web/20200309163509/https:/foodindustry.asia/about/members) | [✓](https://web.archive.org/web/20200309163509/https:/foodindustry.asia/about/members) | [✓](https://web.archive.org/web/20200309163509/https:/foodindustry.asia/about/members) | [✓](https://web.archive.org/web/20200309163509/https:/foodindustry.asia/about/members) |
| Grocery Manufacturers Association (GMA) | U.S.-based grocery trade association. Now called Consumer Brands Association | [✓](https://web.archive.org/web/20200201041651/https:/consumerbrandsassociation.org/membership-benefits/membership-list/) | [✓](https://web.archive.org/web/20200201041651/https:/consumerbrandsassociation.org/membership-benefits/membership-list/) | [✓](https://web.archive.org/web/20131226021050/http:/www.gmaonline.org/forms/MemberDirectory/viewMemberDirectoryAll)^1^ | [✓](https://web.archive.org/web/20200201041651/https:/consumerbrandsassociation.org/membership-benefits/membership-list/) |
| International Council of Beverages Associations (ICBA) | International association representing the non-alcoholic beverage industry, including national and regional associations. | [✓](https://web.archive.org/web/20200309171748/https:/www.icba-net.org/icba-members/icba-members/) | [✓](https://web.archive.org/web/20200309171748/https:/www.icba-net.org/icba-members/icba-members/) | [(✓, through ANDI)](https://web.archive.org/web/20200309171748/https:/www.icba-net.org/icba-members/icba-members/) | [(✓, through ANDI)](https://web.archive.org/web/20200309171748/https:/www.icba-net.org/icba-members/icba-members/) |
| U.S. Council for International Business (USCIB) | U.S.-based general business association focused on international trade. | [✓](https://web.archive.org/web/20200309161338/https:/www.uscib.org/uscib-content/uploads/2018/09/AnnualReport2017_2018_FinalVer_small.pdf) | [✓](https://www.uscib.org/uscib-content/uploads/2018/11/USCIB_AnnualReport2016_2017.pdf)^1^ | x | [(✓, through GMA)](https://web.archive.org/web/20200309161338/https:/www.uscib.org/uscib-content/uploads/2018/09/AnnualReport2017_2018_FinalVer_small.pdf) |
| Alianza Latinoamericana de Asociaciones de la Industria de Alimentos y Bebidas^2^ (ALAIAB) | Latin American food and beverage industry association. | [(✓, through various member BAs)](https://web.archive.org/web/20200309174035/http:/alaiab.org/wp/miembros/) | [(✓, through various member BAs)](https://web.archive.org/web/20200309174035/http:/alaiab.org/wp/miembros/) | [(✓, through various member BAs)](https://web.archive.org/web/20200309174035/http:/alaiab.org/wp/miembros/) | [(✓, through various member BAs)](https://web.archive.org/web/20200309174035/http:/alaiab.org/wp/miembros/) |
| German Federation for Food Law and Food Science (BLL) | German food and beverage industry trade association. | [✓](https://web.archive.org/web/20200309173213/https:/www.lebensmittelverband.de/de/mitglieder/unsere-mitglieder/industrielle-unternehmen/) | x | [✓](https://web.archive.org/web/20200309173213/https:/www.lebensmittelverband.de/de/mitglieder/unsere-mitglieder/industrielle-unternehmen/) | x |
| Federalimentare^2^ | Italian food and beverage industry trade association. | (✓, through [AssoBibe](https://web.archive.org/web/20200309153930/http:/www.assobibe.it/aziende-associate/) & [Italian Food Union](https://web.archive.org/web/20200309154846/https:/www.unioneitalianafood.it/data/file/UnioneItalianaFood-ElencoAziende.pdf)) | (✓, through [AssoBibe](https://web.archive.org/web/20200309153930/http:/www.assobibe.it/aziende-associate/) & [Italian Food Union](https://web.archive.org/web/20200309154846/https:/www.unioneitalianafood.it/data/file/UnioneItalianaFood-ElencoAziende.pdf)) | (✓, through [AssoLatte](https://web.archive.org/web/20200309154411/http:/www.assolatte.it/it/home/chi_siamo/marchi) & [Italian Food Union](https://web.archive.org/web/20200309154846/https:/www.unioneitalianafood.it/data/file/UnioneItalianaFood-ElencoAziende.pdf)) | (✓, through [Italian Food Union](https://web.archive.org/web/20200309154846/https:/www.unioneitalianafood.it/data/file/UnioneItalianaFood-ElencoAziende.pdf)) |
| Engaging America’s Global Leadership^2^ (EAGL) | U.S.-based private sector coalition, focused on promoting U.S. engagement with international organisations. | [(✓, through GMA)](https://web.archive.org/web/20200309184204/http:/americasgloballeadership.org/about-us/) | [(✓, through GMA)](https://web.archive.org/web/20200309184204/http:/americasgloballeadership.org/about-us/) | [(✓, through GMA)](https://web.archive.org/web/20200309184204/http:/americasgloballeadership.org/about-us/) | [(✓, through GMA)](https://web.archive.org/web/20200309184204/http:/americasgloballeadership.org/about-us/) |
| International Special Dietary Foods Industries^2^ (ISDI) | International association representing manufacturers and marketers of special dietary foods. | [(✓, through ABIA)](https://web.archive.org/web/20200309183241/https:/www.isdi.org/about/) | [(✓, through ABIA)](https://web.archive.org/web/20200309183241/https:/www.isdi.org/about/) | [(✓, through ABIA)](https://web.archive.org/web/20200309183241/https:/www.isdi.org/about/) | [(✓, through ABIA)](https://web.archive.org/web/20200309183241/https:/www.isdi.org/about/) |
| Asociación Nacional de Fabricantes de Alimentos y Bebidas (ANFAB) | Ecuadorian food and beverage industry trade association. | [✓](https://web.archive.org/web/20200309175327/https:/anfab.com/wp/sectores/) | [✓](https://web.archive.org/web/20200309175327/https:/anfab.com/wp/sectores/) | [✓](https://web.archive.org/web/20200309175327/https:/anfab.com/wp/sectores/) | [✓](https://web.archive.org/web/20200309175327/https:/anfab.com/wp/sectores/) |
| Australian Food and Grocery Council (AFGC) | Australian food and grocery trade association. | [✓](https://web.archive.org/web/20200226155229/https:/www.afgc.org.au/wp-content/uploads/2019/11/AFGC-Annual-Report-2019-FINAL.pdf) | [✓](https://web.archive.org/web/20200226155229/https:/www.afgc.org.au/wp-content/uploads/2019/11/AFGC-Annual-Report-2019-FINAL.pdf) | [✓](https://web.archive.org/web/20200226155229/https:/www.afgc.org.au/wp-content/uploads/2019/11/AFGC-Annual-Report-2019-FINAL.pdf) | [✓](https://web.archive.org/web/20200226155229/https:/www.afgc.org.au/wp-content/uploads/2019/11/AFGC-Annual-Report-2019-FINAL.pdf) |
| Associação Brasileira da Indústria de Alimentos (ABIA) | Brazilian food industry trade association. | [✓](https://web.archive.org/web/20200309174931/https:/www.abia.org.br/vsn/temp/z2019422RelatorioAnual2018.pdf) | [✓](https://web.archive.org/web/20200309174931/https:/www.abia.org.br/vsn/temp/z2019422RelatorioAnual2018.pdf) | [✓](https://web.archive.org/web/20200309174931/https:/www.abia.org.br/vsn/temp/z2019422RelatorioAnual2018.pdf) | [✓](https://web.archive.org/web/20200309174931/https:/www.abia.org.br/vsn/temp/z2019422RelatorioAnual2018.pdf) |
| Cámara de la Industria de Alimentos de Colombia (ANDI) | Colombian food and beverage industry trade association. | x | [✓](https://web.archive.org/web/20200309173722/http:/www.andi.com.co/Home/Camara/16-industria-de-alimentos) | [✓](https://web.archive.org/web/20200309173722/http:/www.andi.com.co/Home/Camara/16-industria-de-alimentos) | [✓](https://web.archive.org/web/20200309173722/http:/www.andi.com.co/Home/Camara/16-industria-de-alimentos) |
| FoodDrinkEurope | European food and beverage trade association. | [✓](https://web.archive.org/web/20200309163107/https:/www.fooddrinkeurope.eu/about-us/members/) | [✓](https://web.archive.org/web/20200309163107/https:/www.fooddrinkeurope.eu/about-us/members/) | [✓](https://web.archive.org/web/20200309163107/https:/www.fooddrinkeurope.eu/about-us/members/) | [✓](https://web.archive.org/web/20200309163107/https:/www.fooddrinkeurope.eu/about-us/members/) |
| International Sweeteners Association (ISA) | International association representing manufacturers and users of low-calorie sweeteners. | [✓](https://web.archive.org/web/20200309182852/https:/www.sweeteners.org/isa-members) | [✓](https://web.archive.org/web/20200309182852/https:/www.sweeteners.org/isa-members) | x | x |
| Corn Refiners Association (CRA) | US-based trade association representing corn refiners and users of such products (e.g. Tate & Lyle) | x | x | x | x |
| Private Sector Mechanism to the UN Committee on World Food Security (PSM-CFS) | Agri-food private sector platform which holds a seat in the CFS Advisory Group. | [(✓, through ICBA)](https://web.archive.org/web/20200309162551/https:/agrifood.net/private-sector-mechanism) | [✓](https://web.archive.org/web/20200309162551/https:/agrifood.net/private-sector-mechanism) | [✓](https://web.archive.org/web/20200309162551/https:/agrifood.net/private-sector-mechanism) | [(✓, through USCIB)](https://web.archive.org/web/20200309162551/https:/agrifood.net/private-sector-mechanism) |
| Global Dairy Platform | International dairy trade association. | - | - | - | - |
| International Dairy Federation^2 3^ | International dairy trade association with national committees as members. | [✓, one board member from Nestlé](https://www.fil-idf.org/wp-content/uploads/2017/11/IDF-Annual-Report-2017-low-res-30-nov-2017.pdf) | - | - | - |
| AB Chile | Chilean food and beverage industry trade association. | [✓](https://web.archive.org/web/20190604165143/http:/abchile.cl/index.php?page=empresas) | [✓](https://web.archive.org/web/20190604165143/http:/abchile.cl/index.php?page=empresas) | [✓](https://web.archive.org/web/20190604165143/http:/abchile.cl/index.php?page=empresas) | [✓](https://web.archive.org/web/20190604165143/http:/abchile.cl/index.php?page=empresas) |
| ^1^The company was a member of this BA for at least part of the study period (Sept 2015 – Sept 2018) but has left since.  ^2^This is an umbrella association whose members are business associations rather than individual companies; therefore, only second-degree links can be indicated.  ^3^IDF has corporate partners does not share information on who these are (the authors corresponded with IDF). However, a link with Nestlé is indicated as board members include a Senior Executive of the company. | | | | | |
