# Supplementary material for: Non-communicable disease governance in the era of the sustainable development goals: a qualitative analysis of food industry framing in WHO consultations
Source: Global Health. 2020 Aug 26;16:76. doi: 10.1186/s12992-020-00611-1 (PMC7448499; doi:10.1186/s12992-020-00611-1)
Supplement: Supplementary file 2 — Additional file 2. Coding framework used to map positions on policy and preferred governance approaches. This file describes in more detail the framework used to code the policy positions of consultation respondents in our sample. [file 12992_2020_611_MOESM2_ESM.docx]

**Additional file 2: Coding framework used to map positions on policy and preferred governance approaches.**

**Table A2:** Coding framework for positions on policy and governance. If a policy/tool was mentioned, we coded for opposition (policy/tool should not be introduced/included in WHO recommendations), support (policy/tool should be recommended), or neutral (mentioned but no preference expressed, or support for existing rather than new regulation).

| Category | Policy/ tool | Description |
| --- | --- | --- |
| Statutory regulation | Taxation | Fiscal policies aimed at improving public health by reducing consumption of a certain ingredient. In most cases this specifically related to the taxation of sugar-sweetened beverages. |
|  | Mandatory labelling | Introduction of mandatory rules for food and beverage labelling. |
|  | Advertising restrictions | Regulation of unhealthy food and beverage advertising through legislation. |
| Self- & co-regulation | Self-regulation of advertising | Regulation of unhealthy food and beverage advertising through industry-set standards or public-private-initiatives which are not legally binding. |
|  | Voluntary labelling | Rule-setting for food and beverage labelling by industry or through public-private-initiatives which are not legally binding. |
|  | Reformulation | Voluntary reformulation by industry or through public-private-agreements which are not legally binding. |
|  | Education | Promotion of healthy lifestyles through education |
|  | Co-regulation/ PPIs (general) | Public-private-initiatives in which the public sector addresses public health issues by working with the private sector. |
| Governance & policymaking architecture | COI safeguards | Formal safeguards which would restrict the ability of commercial actors to engage in public health nutrition policy. |
|  | Business impact assessment | Impact assessments focused on evaluating the impact of policies on business (economically oriented forms of cost-benefit assessment included here). |
|  | Include industry in reporting | Inclusion of industry in the evaluation and reporting of NCD policy initiatives. |
|  | Broad consultation | Stakeholder consultation during policy development which includes a wide range of actors, notably business. |
