# Supplementary material for: Non-communicable disease governance in the era of the sustainable development goals: a qualitative analysis of food industry framing in WHO consultations
Source: Global Health. 2020 Aug 26;16:76. doi: 10.1186/s12992-020-00611-1 (PMC7448499; doi:10.1186/s12992-020-00611-1)
Supplement: Supplementary file 3 — Additional file 3. Summary of policy positions across all business associations in our sample. This file provides a quantitative overview of how many BAs in our sample supported or opposed a number of policies. [file 12992_2020_611_MOESM3_ESM.docx]

**Additional file 3: Summary of policy positions across all business associations in our sample**

**Table A3:** Positions towards recommending the introduction of the policies/tools listed.

| Category | Policy/ tool | Policy positions expressed by organisations (n=21, AB Chile submission to HLC consultation excluded as faulty) | | |
| --- | --- | --- | --- | --- |
|  |  | *Not explicitly addressed (/no opinion discernible)* | *Supportive* | *Opposed* |
| Statutory regulation | Taxation | 12 | 0 | 10 |
|  | Mandatory labelling | 20 | 0 | 1 |
|  | Advertising restrictions | 17 | 0 | 4 |
| Self- & co-regulation | Self-regulation of advertising | 11 | 10 | 0 |
|  | Voluntary labelling | 14 | 7 | 0 |
|  | Reformulation | 6 | 15 | 0 |
|  | Promoting healthy behaviour through education | 7 | 14 | 0 |
|  | Co-regulation/ PPIs (general) | 0 | 21 | 0 |
| Governance & policymaking architecture | COI safeguards (as proposed) | 15 | 0 | 6 |
|  | Include industry in reporting | 16 | 5 | 0 |
|  | Business impact assessment | 15 | 6 | 0 |
|  | Broad consultation | 11 | 10 | 0 |
